# Epigenetic mechanisms during ageing and neurogenesis as novel therapeutic avenues in human brain disorders

**DOI:** 10.1186/s13148-017-0365-z

**Published:** 2017-06-29

**Authors:** Raúl Delgado-Morales, Roberto Carlos Agís-Balboa, Manel Esteller, María Berdasco

**Affiliations:** 1 Cancer Epigenetics Group, Cancer Epigenetics and Biology Program (PEBC), Bellvitge Biomedical Biomedical Research Institute (IDIBELL), 3rd Floor, Hospital Duran i Reynals, Av. Gran Via 199-203, 08908L’Hospitalet, Barcelona, Catalonia Spain; 2 Department of Psychiatry and Neuropsychology, School for Mental Health and Neuroscience (MHeNs), Maastricht University, Maastricht, The Netherlands; 3 Psychiatric Diseases Research Group, Galicia Sur Health Research Institute, Complexo Hospitalario Universitario de Vigo (CHUVI), SERGAS, CIBERSAM, Vigo, Spain; 4 Department of Physiological Sciences II, School of Medicine, University of Barcelona, Barcelona, Spain; 5 Institució Catalana de Recerca i Estudis Avançats (ICREA), Barcelona, Spain

**Keywords:** Epigenetics, DNA methylation, Histone modifications, Epidrug, Neurogenesis, Neurodegeneration, Psychiatric disorders

## Abstract

Ageing is the main risk factor for human neurological disorders. Among the diverse molecular pathways that govern ageing, epigenetics can guide age-associated decline in part by regulating gene expression and also through the modulation of genomic instability and high-order chromatin architecture. Epigenetic mechanisms are involved in the regulation of neural differentiation as well as in functional processes related to memory consolidation, learning or cognition during healthy lifespan. On the other side of the coin, many neurodegenerative diseases are associated with epigenetic dysregulation. The reversible nature of epigenetic factors and, especially, their role as mediators between the genome and the environment make them exciting candidates as therapeutic targets. Rather than providing a broad description of the pathways epigenetically deregulated in human neurological disorders, in this review, we have focused on the potential use of epigenetic enzymes as druggable targets to ameliorate neural decline during normal ageing and especially in neurological disorders. We will firstly discuss recent progress that supports a key role of epigenetic regulation during healthy ageing with an emphasis on the role of epigenetic regulation in adult neurogenesis. Then, we will focus on epigenetic alterations associated with ageing-related human disorders of the central nervous system. We will discuss examples in the context of psychiatric disorders, including schizophrenia and posttraumatic stress disorders, and also dementia or Alzheimer’s disease as the most frequent neurodegenerative disease. Finally, methodological limitations and future perspectives are discussed.

## Background

Ageing, defined as the progressive functional decline of organisms at molecular, cellular and physiological level, is the main risk factor for major human diseases such as cancer, cardiovascular diseases or neurological disorders [1]. As a part of natural ageing, the human brain and nervous system go through natural changes that result in neuronal death and decline of memory, cognitive and coordination processes, among other functional impairments. The effects of ageing on the central nervous system are widespread, have multiple aetiologies and have different clinical manifestations depending on the person.

We must highlight that age-associated decline is part of the natural lifespan; however, this loss of neural function can also be associated with pathogenic conditions in a broad range of human disorders, including neurodevelopmental diseases (e.g. Rett syndrome), neurodegenerative disorders (dementia, Alzheimer’s disease, Parkinson’s disease, amyotrophic lateral sclerosis, etc.) or changes in behaviour leading to psychiatric diseases. Most of these complex disorders are the result of alterations in multiple molecular pathways together with the interaction of environmental factors.

It is clear that accumulating evidence of how these ageing-associated processes occur at molecular level will provide promising “druggable” targets for therapy in ageing-related disorders. In this way, much attention is paid to the molecular basis of ageing using many experimental cellular contexts, such as telomere shortening, DNA damage, loss of proteostasis and degeneration of cell or organ structures [1]. Nowadays, it is also widely accepted that changes in epigenetic modifications are a phenomenon associated with ageing throughout the control of gene expression and genomic instability [2, 3]. The dynamic and reversible nature of epigenetic alterations makes epigenetic mechanisms optimal targets for the development of novel treatment strategies in neurological disorders, a strategy that is currently used in the clinical management of other human complex disorders such as cancer [4].

In this review, we will summarize our current knowledge about the involvement of epigenetic factors in normal ageing (ageing-associated epigenome) and those environmental factors influencing the epigenetic landscape of an organism and that can be more easily modified with lifestyle (e.g. diet, stress or smoking). Since the use of agents and manipulations that boost neurogenesis is an important strategy to improve neurological function in human disorders with neural decline, we will also summarize the current uses of epigenetic-based treatments to improve adult neurogenesis. Additionally, we examine the preclinical studies about the use of pharmacological treatments to reverse the epigenetic signature and ameliorate neural dysfunction in human disorders, including common psychiatric disorders (schizophrenia and posttraumatic stress disorder), dementia and the most well-known neurological disorder, Alzheimer’s disease. In recent years, a lot of attention has been paid to the role of non-coding RNAs (ncRNAs) in the neural differentiation processes but also in the ethiopathology of neurological disorders [5]. However, due to current lack of ncRNA-based therapeutic strategies, we will focus on evidence accumulated with treatments targeting DNA methylation (and DNA demethylation) and histone modifications. In most cases, especially in psychiatric disorders, knowledge is still in its infancy and many questions about the epigenetic basis underlying the disease are yet to be addressed. Neurodegenerative diseases are a complex heterogeneous group of diseases, and the comprehensive understanding of the mechanisms involved in their initiation and progress should overpass some limitations in the research strategies. Some improvements are still needed, including increased sample size of the cohorts, more appropriate animal models for the diseases, multicentric validations or multivariable analysis. Elucidating the epigenetic signatures of brain diseases is imperative to developing and applying epigenetics-driven therapeutic approaches.

## Main text

### Age-related epigenetics

Studies of the epigenome have outlined a chromatin signature during human normal ageing. It is described that there is a general loss of histones [6] together with a massive alteration in the histone modification patterns. The global trends of the ageing- associated histone code are a loss of repressive marks and a gain of activating transcriptional marks, both actions resulting in gain and loss of heterochromatin regions. As examples, redistribution of the active histone mark H3K4me3 over tissue-specific genes [7] or gain of H4K16ac and H3K56ac [8] are hallmarks of ageing. As a consequence of the histone switch, widespread transcriptional deregulation occurs that includes global amplification of canonical transcripts [2]. Additionally, there are global and local changes of the methylome during mammalian ageing [9, 10]. Decreased CpG methylation was found in advanced aged blood samples, mainly affecting methylation spots into enhancers. In contrast, gain of methylation was also observed at specific loci at CpG islands [10], and interestingly in loci near tissue-specific genes, genes coding for transcription factors or genes associated with differentiation and development [2]. An important consideration for the role of CpG methylation in ageing is its tissue specificity, since methylation loci can vary from one cell type to another. In spite of potential divergences among tissues should be considered, a slow and gradual loss of genome-wide methylation (global hypomethylation) together with gain of methylation at specific loci (specific hypermethylation) during ageing was also reported in brain human tissues [11]. In summary, the altered pattern of CpG methylation during one’s lifespan is congruent with the changes in histone modifications marks at specific transcriptional networks. It remains to be elucidated what the roles of these specific genes are in the ageing process.

The relation between the effect of genetic variation and epigenetics should also be considered. The genetics underlying longevity have been widely explored [12, 13], but non-genetic contribution can be a confounding factor in these studies. Monozygotic twins are exceptional models for assessing the epigenetic effects of ageing on identical genomes [14, 15]. These studies showed that the epigenetic discordance between twins increased with ageing and support the idea that epigenetic drift is overcome by environmental factors during lifespan. Finally, it is important to consider that at present, it is unclear whether changes in epigenetic marks altered the expression of genes associated with ageing or whether the disturbance of molecular pathways during ageing results in epigenetic changes at higher scales in the genome. In other words, it is still unknown if epigenetic changes are drivers or just consequence of the ageing process. Recent technological advances provide useful tools for addressing these challenges, such as the use of CRISPR/dCas9 for enzyme targeting. In particular, epigenetic editing for rewriting the epigenome at specific loci will greatly contribute to the deciphering of the causative versus correlative changes in ageing [16].

### Epigenetic as a bridge between environmental signals and genome response during early life and ageing

**Fig. 1:**
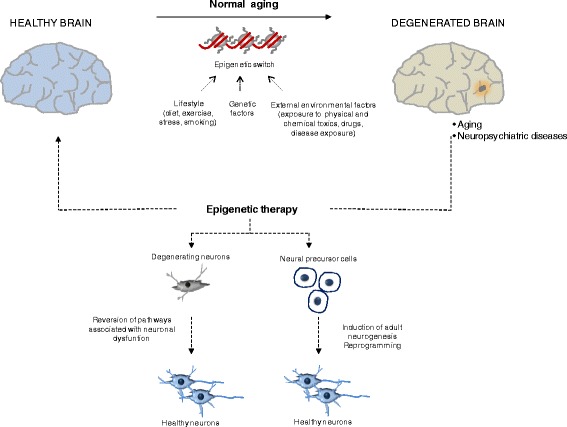
Epigenetic therapy in neuropsychiatric disorders. A combination of external and internal factors can induce epigenetic changes in the normal healthy brain during ageing but also under pathological conditions, including neurodegenerative disorders or psychiatric diseases. Epigenetic therapy, such as pharmacological treatment with DNA-demethylating drugs or HDAC inhibitors, can reverse the distorted epigenetic modifications and induce specific gene expression programs. Two main strategies can be achieved: first, the use of epigenetic drugs can induce adult neurogenesis by enhancing cellular reprogramming from neural precursor cells and, second, changes in epigenetic modifications can reverse gene expression of genes involved in neuronal dysfunction and relevant for the disease

Internal and external environmental factors that are well-known contributors of ageing can be integrated into genome response by means of epigenetic responses (Fig. 1). Alterations in epigenetic modifications can be associated with caloric restriction, lower basal metabolic rate or stress, among others [17]. An increasing number of studies on the influence of the environment during in utero and in early stages of development have provided evidence of how external stimuli during stages of early life, such as exposure to toxins or nutritional deficiencies, govern the extent of disease vulnerability [18]. It has been proposed that environmental factors may interact with specific loci thereby modifying their expression through epigenetic mechanisms and increasing disease susceptibility in later life [19]. The effect of fetal nutrition, which depends on nourishment provided by the maternal system, has been widely described both in animal and human models [19, 20], and a positive relation between maternal diet and neurodegeneration has been supported in some human studies. The offspring of women exposed to the Dutch famine in 1944–1945 had significantly increased risk of several disorders, including schizophrenia [21, 22]. Although several components of the diet can mediate this effect, an association between vitamin B12 and folic acid supplementation in the mother’s diet during pregnancy and defects on the myelination in the nervous system of offspring has been described [23]. In a similar manner, the negative effect of fetal exposure to factors such as tobacco, alcohol consumption, stress or infections had also been investigated in the context of risk to neurological disorders in the offspring [24–26]. How epigenetics modulate changes in brain development and functions even across generations (the named “transgenerational inheritance”) will be the subject of future research in the field and surely will contribute to improve strategies supporting healthy development.

It must be considered that embryonic development is the most susceptible period due to the high number of cell replication events and epigenetic drifts that take place during differentiation [18]. In postnatal development, several observations can also support the environment- epigenome connection. Diet is probably the best example of external stimuli affecting the epigenome and the ageing phenotype. Restrictions in nutrient intake (below the levels of malnutrition) extend lifespan and delay ageing in many organisms from yeast to humans [27]. One of the major factors activated under conditions of caloric restriction is Histone Deacetylase (HDAC) protein Sirtuin 1 (SIRT1) [3, 28]. The beneficial effects of activation of SIRT1 are exerted, at least in part, by improving mitochondrial function [29], but also because SIRT1 expression after caloric restriction results in decreased levels of inflammation-associated genes [28]. The activity of the sirtuin family of histone deacetylates is dependent on the cofactor NAD+ and NAD+ levels decline with age. Modulation of NAD+ availability, for example as a result of NAD+ donors in the diet, can result in altered SIRT1 function [30] and contrasts ageing effects. High NAD+ levels are also a consequence of an oxidative metabolic state induced by caloric restriction [3] and ameliorate ageing. Caloric restriction has been also associated with 5-methylcytosine contents and Dnmt3 activity in the hippocampus of mice models of ageing [31]. Similarly, it has also been shown that high nutrient intake mimics the CpG methylation profile of ageing cells in the liver [32].

Life stress has been also associated with health span and longevity and is a risk factor for late-life neurological and metabolic disorders [17]. Telomere shortening has been described in association with adult stress conditions [33]. In addition, life stressors induce alterations in CpG methylation in early stages of development in humans, affecting specific genes such as glucocorticoid stress response-mediators [34, 35]. Glucocorticoids can exert two complementary actions to decrease CpG methylation. On the one hand, they decrease the expression of the DNA methyltransferase DNMT1 in neurons [36], and on the other hand, exposure to glucocorticoids may upregulate the expression of DNA demethylases from the ten-eleven translocation (TET) family [37].

Healthy and non-healthy habits can also ameliorate or accelerate, respectively, ageing. Little is known about the underlying molecular mechanisms of the beneficial effect of exercise during ageing, but a few reports about the role of epigenetics exist. Decreased levels of the histone deacetylases HDAC4 and HDAC5 and increased levels of acetylated H3K36 were detected in human skeletal muscle in the brain after exercise [38, 39]. On the other hand, tobacco exposure can alter the CpG methylation status of genes associated with cellular homeostasis and development of ageing-associated disorders [40]. Furthermore, decreased H4K16Ac and increased H3K27me3 levels at specific locus have been also described in smokers [41]. Alcohol inhibition of the methionine synthase pathway, and consequently the S-adenosylmethionine levels, may be associated with hypomethylation of LINE sequences related to alcohol consumption [42].

Again, causal evidence that can directly link environmental factors and specific chromatin reordering through epigenetic mechanisms, and in consequence, affect ageing and health span, is still missing. Further research will open new avenues of chromatin-based strategies to delay (or even reverse) ageing and ageing-related diseases by manipulation of lifestyle conditions.

### Epigenetic contributions to neurogenesis induction during ageing

One of the main end-point manifestations associated with ageing is loss of neuronal function that leads to impairment of memory and cognition. As aforementioned, epigenetic alterations contribute to the natural process of “healthy” ageing; however, the reversible nature of epigenetic marks adds extra value to them as potential targets for ameliorating neurological decline during ageing.

Although still far from clinical use, improving adult neurogenesis is a promising strategy to treat neurological disorders (Fig. 1). In the adult vertebrate brain, the formation of new neurons takes place in a specific population of cells referred as neural stem progenitor cells (NSPCs). Neurogenesis is generally not a frequent process under normal physiological conditions, but it is described to be induced after brain injury [43]. NSPCs can be found in local niches of the brain, such as the subventricular zone (SVZ) of the lateral ventricle or the subgranular zone (SGZ) of the dentate gyrus (DG) of the hippocampus [44]. Neurogenesis at SGZ has received a lot of attention due to its involvement in cognitive functions such as memory consolidation. Granule cells, the providers of excitatory input to the pyramidal cells of CA3 region, are the unique type of neuron that is generated from the NSPCs in the SGZ under physiological conditions [43]. Although it is still unclear how intrinsic and extrinsic mechanisms induce adult neurogenesis, different signals have been identified including presence of specific cytoplasmatic factors (growth factors, neurotrophins, cytokines and hormones, among others), transcriptional factor network and epigenetic regulators [44, 45].

In recent years, further evidence has demonstrated the role of epigenetic factors in the maintenance of neural stem cell renewal and also in the induction of new mature neurons. Although the contribution of ncRNAs to neural differentiation has been reported in different experimental systems, and especially for microRNAs, knowledge about their functional relevance is still in its infancy [46] and their therapeutic potential is largely unexplored. By contrast, the role of CpG methylation and histone modifications in neuronal cell fate and stem cell self-renewal has been widely explored [47–50]. For example, well-known pluripotency genes are epigenetically inactivated by CpG promoter methylation during adult neurogenesis from NSPCs [47]. Interestingly, changes in DNA methylation as a consequence of external stimuli and promotion of adult neurogenesis have been described. Physical exercise can induce neurogenesis, and during this process, an association with changes in the promoter methylation of the neurotrophic factor BDNF [48] were described. Loss of demethylation by active mechanisms should also be considered, such as the activation of the GADD45B demethylase in the DG cells during adult neurogenesis [49] or the transformation of 5-methylcytosine (5-mC) into 5-hydroxymethylcytosine (5-hmC) by enzymes of the TET family [50]. 5-hmC is enriched in adult neurons compared to NSPCs of the SVZ in the mouse developing brain and colocalize with MeCP2 and with the active chromatin histone modification H3K4me2 in mouse neurons [51]. Another mechanism by which CpG methylation results in transcriptional silence is by binding to methyl-CpG-binding proteins that recruit several chromatin remodelling proteins. As an example, it has been described that the Methyl-CpG-Binding Domain Protein 1 (MBD1) suppresses the expression of FGF-2 promoting differentiation during adult neurogenesis in the hippocampus [52]. MECP2 encodes an epigenetic factor that influences chromatin structure and considered to act mainly as a transcriptional repressor [53]. Furthermore, recent studies using induced pluripotent stem cells derived from Rett patients (a disorder causes generally by point mutations on the MECP2 gene) demonstrated the role of the MeCP2 protein in neuronal maturation [54]. In addition to DNA methylation, histone modifiers serve as important regulators in neuronal development. Mll1 (mixed-lineage leukaemia 1) is a histone methyltransferase (HMT) that is required for neuronal differentiation in the adult SVZ and its effect can be exerted by regulating the expression of DLX2 by increasing H3K27 methylation [55]. Histone acetylation-related enzymes, such as HDAC2, also impact the maturation and survival of adult neurons in the SVZ region [56].

The idea of effective stimulation of neuronal production by using epidrugs is highly attractive, and although in its infancy, it is supported by several lines of evidence (Table 1). Interestingly, pharmacological inhibition of HDAC activity alters neuronal differentiation. It has been reported that treatments with trichostatin A (TSA) or valproic acid (VPA) induced neuronal differentiation in adult progenitor cells [46, 57]. VPA treatment also improved the differentiation of sympathoadrenal progenitor cells into catecholaminergic neurons [58]. Epigenetic drugs targeting histone methylation are less extensively addressed. Pre-administration of Bix-01294, a G9a/GLP inhibitor, has a neuroprotective effect in a mouse model of neurodegeneration induced by ethanol and prevents deficits in long-term potentiation, memory and social recognition behaviour [59]. The underlying molecular mechanisms are still unclear although reactivation of specific genes involved in cell fate after epidrug treatments has been identified. For instance, TSA treatment of the PC12 cell line results in increased acetylation of Lys14 on histone H3 and upregulation of the expression of nur77 gene [60]. A neuroprotective effect of HDAC inhibitor (HDACi) treatment mediated by inflammation prevention has been also suggested [61]. It must be highlighted that multitargeting is also possible after treatment with epigenetic-based drugs due to the lack of isoform selectivity and also due to the off-target effects affecting non-histone proteins. As an example, treatment with the HDACi AR-42 restores the abnormalities in histone 4 acetylation observed in an in vitro model of Kabuki syndrome (with mutations in the KMT2D histone methyltransferase) and also alters methylation at H3K4 [62]. Pharmacological manipulation of chromatin complexes is also an alternative. The histone-interacting BET bromodomain proteins are downregulated during neurogenesis from NPCs, and the use of a bromodomain selective inhibitor (JQ-1) results in an increase in neuronal differentiation [63].

**Table 1:** Epigenetic-based treatments associated with manipulation of neurogenesis in mammals

Experimental system	Organism	Epigenetic drug	Functional effect	Ref.
Neural progenitor cells	Rat	VPA	Promotion of neuronal fate, inhibition of glial fate	[46]
Adrenomedullary sympathoadrenal progenitors	Bovine	VPA	Promotion of catecholaminergic neuronal differentiation	[58]
Brain neuroblastoma	Mouse	TSA	Induction of neurite extension	[201]
Cerebella granule neurons	Rat	TSA	Promotion of neuronal outgrowth	[202]
Adrenal medulla progenitors	Rat	TSA, VPA	Induction of neurite outgrowth	[60]
Neural progenitor cells	Mouse	JQ-1	Promotion of neurogenesis, inhibition of gliogenesis	[63]
Neural progenitor cells	Mouse	SAHA, TSA	Reduction in neurogenesis in the ganglionic eminences, increase in neurogenesis in the cortex	[203]
Primary glial cultures and glioblastoma cells	Human	VPA	Alteration of glial cell morphology	[204]
Neural stem cells	Rat	5-AZA	Reduction of migrated neurons and differentiation	[205]

Representative examples of studies are included. *5-AZA* 5-aza-cytidine, *SAHA* suberoylanilide hydroxamic acid, *TSA* trichostatin A, *VPA* valproic acid

### Therapeutic epigenetic-driven approaches to treat psychiatric disorders

Epigenetic disequilibria could influence neurodevelopment and brain function at the level of neural circuits and behavioural outcome and be the trigger point of several psychiatric disorders [64–66] (Table 2). It is well known that genetic and environmental factors contribute to the underlying cause of schizophrenia (SZ) [66–73]. Recently, it was shown that the mammalian brain suffers a global epigenomic reconfiguration during fetal to young adult development which could influence SZ onset specifically before the age of 20 [11]. Epigenetic processes can be developmentally regulated, altered by drugs and environmental factors, and be tissue-specific [65, 66, 71, 74] and provide links between clinical manifestations of the psychiatric phenotype and lifestyle. For example, inhalation of nicotine by tobacco smoking (a confounding factor), regularly practised by SZ patients, could work as a self-medication. It is proposed to correct a cholinergic (nicotinic) neurotransmission deficit in those patients via epigenetic actions on GABAergic neurons [71, 75].

**Table 2:** Epigenetic dysregulation in schizophrenia, PTSD (post-traumatic stress disorders) and Alzheimer’s disease

Disorder	Gene	Molecular effect	Specie	Method	References
Schizophrenia					
EPHA4, PKNOX1, ESR1, among others		DNA methylation, hsa-miR-219a-5p	Human	Gene set enrichment analysis	[206]
NUBP1, PRKCE, HLA-DQA1, HLA-B, FRK, IL12RB1, among others		DNA methylation	Human	DNA methylation array	[74]
FAM63B, among others		DNA methylation	Human	Methylome-wide association study, targeted pyrosequencing of bisulfite-converted DNA	[207]
GAD67		DNA methylation, H3 acetylation	Mouse	MeDIP, ChIP, qPCR, Western Blotting	[75, 77, 85]
REELIN		DNA methylation, H3 acetylation	Mouse, Human	MeDIP, ChIP, qPCR, Western Blotting, Gel Shift Binding Assays, Methylome-wide association study, targeted pyrosequencing of bisulfite-converted DNA	[77, 85, 207, 208]
BDNF		DNA methylation, H3 acetylation	Mouse, Human	MeDIP, ChIP, qPCR, Microarray-based DNA methylation profiling	[76, 209]
mGlu2		DNA methylation, H3 acetylation	Mouse	Bisulfite sequencing, ChIP, qPCR	[210]
HLA genes, among others		Histone acetylation	Mouse	Microarray studies, qRT-PCR, Western Blotting	[114]
COMT		DNA methylation	Human	Bisulfite, DNA sequencing, Methylated SpecificPCR and bisulfite sequencing, qRT-PCR	[211, 212]
S-COMT		DNA methylation	Human	Pyrosequencing	[213]
FOSP2		DNA methylation	Human	Bisulfite DNA sequencing	[214]
HTR2A		DNA methylation	Human	Bisulfite DNA sequencing	[215]
SOX10		DNA methylation	Human	Bisulfite DNA sequencing	[216]
5HTR1A		DNA methylation	Human	High-resolution melt assay	[217]
MAOA		DNA methylation	Human	Methylated Specific PCR	[218]
MEK1		DNA methylation	Human	Microarray-based DNA methylation profiling	[219]
CAMKIIγ		miR-129	Mouse	miRNA array profiling, miRNA specific RT-PCR	[220]
PTSD					
BDNF		H4 acetylation	Mouse, Rat	Western Blotting, ChIP, qPCR	[221, 222]
FKBP5		DNA methylation/hydroxymethylation	Mouse	Bisulfite sequencing, Pyrosequencing	[34, 223]
HDAC1, cFos		H3 acetylation and methylation	Mouse	ChIP, qPCR	[224]
NR2B		H3 and H4 acetylation	Rat	Western Blotting, ChIP, RT-PCR	[225]
CBP, p300, PCAF		H2B and H4 acetylation	Rat	Western Blotting, ChIP, PCR	[226]
Calcineurin (CaN)		DNA methylation	Rat	Bisulfite sequencing	[227]
PP1		DNA methylation	Rat	DNA methylation assay	[228]
REELIN		DNA demethylation	Rat	DNA methylation assay	[228]
IGF2, cFOS, ARC		H3 acetylation	Mouse	ChIP, qPCR	[229]
TLR1, IL8, CNTN2, among others		DNA methylation	Human	DNA methylation array	[230, 231]
MAN2C1		DNA methylation	Human	DNA methylation array	[232]
IGF2, H19, IL8, IL16, IL18		DNA methylation	Human	Pyrosequencing	[233]
COMT		DNA methylation	Human	DNA methylation array	[234]
ADCYAP1R1		DNA methylation	Human	DNA methylation array	[235]
NR3C1		DNA methylation	Human	Pyrosequencing and clonal sequencing	[236–238]
SLC6A3		DNA methylation	Human	DNA methylation array	[239]
SLC6A4		DNA methylation	Human	DNA methylation array	[240]
APC5, TPR, CLEC9A, ANXA2, TLR8		DNA methylation	Human	DNA methylation array	[241]
FKBP5		miR-511	Mouse	qPCR	[242]
Alzheimer’s disease					
APP		DNA methylation	Human	MSRE-SB, pyrosequencing	[125–127]
BACE1		DNA methylation	Cell lines	HPLC	[129]
PSEN1		DNA methylation	Cell lines	HPLC	[129]
Neprylisin		DNA methylation	Cell lines	MS-PCR	[131]
DUSP22		DNA methylation	Human	BS-array, pyrosequencing	[117]
DUSP22		DNA hydroxymethylation	Human	WG 5-hmC-enriched seq	[117]
SORBS3		DNA methylation	Human	BS-array, pyrosequencing, MS-PCR	[116, 132]
NF-kB		DNA methylation	Human and Cell lines	Specific Methylation Assay	[133, 134]
COX2		DNA methylation	Human and Cell lines	Specific Methylation Assay	[134]
BDNF		DNA methylation	Human	MSRE-PCR	[133]
CREB		DNA methylation	Human	MSRE-PCR	[133]
TBXA2R		DNA methylation	Human	BS-array, pyrosequencing, MS-PCR	[116]
ANK1		DNA methylation	Human	WGBS, BS-array, pyrosequencing	[121, 122]
BACE1		H3 acetylation	Human	FAIRE/ChIP	[130]
Neprylisin		H4 acetylation	Cell lines	ChIP	[144]
miR-9		downregulation	Human	PCR	[179]
miR-26a		upregulation	Human	PCR	[161]
miR-29a/b-1		downregulation	Human	miRNA microarray	[164]
miR-29c		downregulation	Mouse	RT-PCR	[164, 168]
miR-34c		downregulation	Human	RNA sequencing	[178]
miR-101		downregulation	Cell lines	PCR	[158]
miR-106b		downregulation	Human	miRNA microarray, Northern blot	[157]
miR-107		downregulation	Human	miRNA microarray	[163]
miR-124		downregulation	Human	RT-PCR	[160]
miR-125		upregulation	Human	Northern blot	[161, 174, 183]
miR-132		downregulation	Human	Northern blot	[161, 174, 183]
miR-137		downregulation	Human	PCR	[162]
miR-153		downregulation	Human	PCR	[158]
miR-181c		downregulation	Human	PCR	[161, 162, 179]
miR-132		downregulation	Human	Northern blot	[174]
miiR-219		downregulation	Human	RNA sequencing	[170]
miR-339-5p		downregulation	Human and Cell lines	RT-PCR	[167]
BACE1-AS		upregulation	Human and Cell lines	RT-PCR	[169]
lnc-17A		upregulation	Human	RT-PCR	[184]

DNA methylation, histone alterations and microRNA expression is summarized. *BS-array* bisulfite-modified DNA based arrays, *ChIP* chromatin immunoprecipitation, *FAIRE* formaldehyde-assisted isolation of regulatory elements, *HPLC* high-performance liquid chromatography, *MeDIP* methylated DNA immunoprecipitation, *MS-PCR* methylation specific PCR, *MSRE-PCR* methylation-sensitive restriction enzyme-PCR, *qPCR* quantitative real-time PCR, *WG 5-hmC-enriched seq* whole-genome sequencing analysis of 5-hydroxymethylcytosine-enriched sequences, *WGBS* whole-genome bisulphite sequencing

It is also remarkable that pathways relevant for actual therapeutic management of SZ are regulated by epigenetic mechanisms. Clinically, the main antipsychotic treatments target the dopaminergic, serotoninergic and monoaminergic receptor systems that exert therapeutic effects in SZ patients [67]. The aetiology of SZ and lifetime antipsychotic use has been associated with DNA methylation changes in MEK1 gene promoter in the frontal cortex of the post-mortem brain. Around 30% of people with SZ have treatment-resistant SZ, and in this case, clozapine is the only effective treatment [73]. In mice, GADD45b mRNA is increased by clozapine [76], but not haloperidol, via stimulation of H3K9 acetylation [77]. Clozapine may exert its therapeutic actions by acting on GABAergic and glutamatergic gene promoters [76, 78, 79], in part targeting DNA methylation via GADD45b, as well as histone methylation and chromatin relaxation [6].

Research during the last two decades suggested that abnormal RELN, DNMT1 and glutamic acid decarboxylase 67 (GAD67) neuronal expression are a feature observed in animal and human brains of SZ patients [71, 80]. Human post-mortem studies show that RELN is downregulated in GABAergic neurons of psychotic patients due to promoter hypermethylation of RELN gene that is associated with an increase in DNMT1 and DNMT3a which is consistent with the “epigenetic GABAergic theory of major psychosis” [71, 80]. Interestingly, early life stress can impact the methylation levels of selected promoters; accordingly, behavioural programming is possible and potentially reversible, at least, in animal models [81]. In a mouse model of prenatal restraint stress that induces epigenetic remodelling in offspring, clozapine but not haloperidol reduces the increased DNMT1 and TET1 levels in frontal cortex of adult prenatal restraint stress mice and also reduces DNMT1 binding to RELN, GAD67 and BDNF promoters [82].

In SZ, HDACis also have a lot of potential as pharmacological treatments. In this context, histone H3 phosphorylation is increased in peripheral blood mononuclear cells when compared to healthy controls [83]. It is known that some HDACis facilitate DNA demethylation [84]. Moreover, combinations of various antipsychotics (e.g. clozapine, olanzapine, quetiapine) and valproic acid (VPA), a HDACi that reduces MeCP2 binding to RELN and GAD67 promoters [85], might prove beneficial in the treatment of SZ based on results from animal and clinical studies [73].

Lastly, disequilibria in microglia and mitochondrial function must also be taken into consideration when discussing SZ. Microglia is important for immune defence in the central nervous system, and the HDACi butyrate influences microglial function and has potential therapeutic functions in SZ [86]. It is known that butyrate among other functions in the CNS reinforces memory function [87] and synaptic plasticity [64, 88]. Mitochondrial dysfunction and cellular energy dysfunction are also associated with SZ. In this context, the butyrate and several carnitinoids could have potential as therapeutic agents to treat SZ and other brain disorders [88].

Posttraumatic stress disorder (PTSD) can develop as a result of a terrifying and traumatic event that can have short-term or long-lasting effects on neuronal function, brain plasticity and behavioural adaptations to psychosocial stressors [89, 90]. Excessive fear and anxiety are some of the main hallmarks of PTSD where extinction training leads to a gradual reduction of fear called “fear extinction” in animals and “exposure-based therapy” in humans. This extinction mechanism and its molecular mechanisms are well conserved across species [90–92]. Considerable progress has recently been made in the preclinical development of cognitive enhancers (e.g. D-cycloserine, yohimbine and glucocorticoids) that potentiate fear extinction. As a result, several targets had been identified, including diverse neurotransmitter systems but also proteins from the IGF2, BDNF and FGF2 pathway or epigenetic modifications and their downstream signalling pathways [90, 91]. The PTSD phenotype is complex and, as many other brain diseases, emerges from interactions between multiple genetic and epigenetic factors [89, 90]. We will focus on the most-well studied epigenetic modifications in fear extinction, DNA methylation and acetylation of histone proteins. However, the importance of ncRNAs in post-transcriptional regulation of gene expression in PTSD is well established [89, 93, 94]. It can be mentioned as an example the role of miR-138b that inhibits the original fear memory and downregulates plasticity-related genes (e.g. creb1 and sp1) in the infralimbic prefrontal cortex of mice [95]. Based on current literature, successful fear extinction is mediated by epigenetic mechanisms, which include enhancement of HAT activity, reduction of HDAC activity (e.g. HDAC2), DNA methylation and DNA demethylation by TET proteins [89, 90]. There are several animal and human studies supporting the role of DNA methylation in PTSD [89]. In mice, for example, DNA methylation is increased in BDNF exon IV in females resistant to fear extinction, which leads to a decrease in BDNF expression in the prefrontal cortex [96]. Interestingly, it has been demonstrated in mice that IGF2/IGFBP7 signalling regulates fear extinction via an upregulation of IGF2 and downregulation of IGFBP7, which promotes survival of 17–19-day-old newborn hippocampal neurons [97]. Both IGF2 and IGFBP7 genes are regulated via DNA methylation and other epigenetic mechanisms [91, 98]. This signalling pathway might have potential as therapeutic target for PTSD, although this possibility will need to be studied further.

In humans, several genes associated with stress response (e.g. NR3C1, FKBP5), neurotransmitter activity (e.g. SLC6A4), immune regulation (e.g. IGF2, H19, IL8, IL16, IL18) and repetitive genomic elements (e.g. LINE-1, Alu) were studied in peripheral blood using either a candidate genetic locus or a genome-wide approach. It was found that their methylation levels are altered in PTSD patients [89]. The TET family of methylcytosine dioxygenases enzymes (TET1, TET2 and TET3) undergoes DNA demethylation (i.e. reverses DNA methylation), which seems to also play an important role during fear extinction [99]. One recent study demonstrates that gene knockdown of TET1 impairs extinction [100]. Furthermore, another study shows that 5-hmC and TET3 occupancy undergo genome-wide redistribution on extinction-related genes and that Gephyrin mRNA expression is increased in the infralimbic prefrontal cortex [101]. These preclinical findings have made TET enzymes and DNA demethylation promising therapeutic targets to potentiate fear extinction; however, compounds that target the TET enzymes and subsequently DNA demethylation are not yet available.

SPV106, a HAT p300/CBP-associated factor (PCAF) activator, facilitates fear extinction and protects against fear renewal when injected in rodent infralimbic prefrontal cortex [102]. On the other hand, if we inhibit HAT p300 in the infralimbic cortex, strengthen fear extinction is enhanced [103]. Thus, HAT modulators affect fear extinction in different ways, and additional work is required to unravel their mechanisms of action. Alternatively, gene transcription of extinction-relevant genes that can be enhanced by HDAC inhibitors such as TSA, sodium butyrate, entinostat (MS-275), vorinostat (SAHA), VPA and Cl-944 can strengthen fear extinction displaying better selectivity towards class-I HDACs [89, 90]. In this context, targeting specific HDAC isoforms could be a useful therapeutic approach to modulating fear extinction [104]. Moreover, MS-275, SAHA and Cl-944 rescue fear extinction deficits in various rodent models [89, 90], and HDAC2 seems to play a crucial function in this rescue [105]. Bahari-Javan et al. observed in rodents that HDAC1 is required for extinction learning that comprises H3K9 deacetylation followed by H3K9 trimethylation of target genes [92, 106]. These facilitating effects on fear extinction are likely due to initiation of various extinction-related gene transcription programs. For example, SAHA and VPA increase acetylation in the promoter of GRIND2B (NMDA receptor subunit 2B) and histone H4 acetylation in the promoter IV of BDNF; Cl-994 increases histone H3 acetylation in the promoter region of plasticity associated genes (e.g. IGF2, ARC, C-FOX), and some neurotransmitter systems increase H3 acetylation in the promoter of certain genes (e.g. BDNF, CAMK2A, CREB) [90, 105].

### Epigenetic link between psychiatric disorders and dementia

Gene expression in the human brain changes with age [106], and it is known that some psychiatric disorders (e.g. SZ, PTSD and depression) may trigger or accelerate the progression of dementia, a neurodegenerative disease [92, 107–109]. Although the specific molecular link has not been properly established, epigenetic effects on disease phenotypes may explain how early life stressors (e.g. a psychiatric disorder) can account for the susceptibility of dementia later in life [11]. For example, in this context, there is evidence showing that childhood abuse induces differential DNA methylation and gene expression patterns in PTSD patients compared to PTSD patients without childhood abuse [110]. IGFBP7, one of the seven IGFBPs identified in the mammalian genome that is used to transport and regulate the bioavailability of IGF1 and IGF2, is deregulated in PTSD and dementia via *Igfbp7* promoter DNA methylation in mouse and human brains [91, 98]. In this context, epigenetic pharmacology emerges as an appealing alternative to treat multifactorial diseases with deregulation in multiple signalling pathways in the framework of personalized medicine [111].

Epigenetic mechanisms are essential for normal brain function (i.e. learning and memory processes), and on the basis of the literature presented in this review, disruptions of these mechanisms are closely related to the molecular alterations associated with disorders such as depression or Alzheimer’s disease. Alzheimer’s disease (AD) is the most prevalent form of dementia currently affecting more than 48 million people worldwide with devastating consequences for the affected patients, their relatives and health systems. Its incidence is expected to double every 20 years (from 74.7 million in 2030 to 131.5 million in 2050, according to the World Alzheimer Report 2015). Increasing life expectancy calls for the urgent development of strategies to delay, attenuate or prevent Alzheimer’s disease, since therapeutic approaches directed only at the moderate-to-late stages have been disappointing in clinical trials so far. AD is divided in two subtypes: familial and sporadic cases. Familial AD (FAD) accounts for only approximately 5% of all AD cases and is associated with inherited mutations in the amyloid precursor protein (APP) gene and the presenilin 1 and 2 (PSEN1/2) genes [112]. This subtype is characterized by presentation of the major hallmarks of the disease before the age of 55 years (early onset AD (EOAD)). Sporadic cases of AD usually present a later age of onset (≥65 years; late onset AD (LOAD)), and the scientific community has still not associated this subtype to any genetic mutation. Thus, identification of non-genetic factors that trigger sporadic forms of the disease is crucial in terms of prevention, and knowledge of the underlying etiopathogenic mechanisms will aid the development of timely interventions.

### Epigenetic deregulation in neurodegenerative disorders: Alzheimer’s disease as a model

Since recent studies have described that gene–environment interactions may underlie neuropsychiatric disorders [113–115], many research efforts have been oriented into the study of the alterations of the “physiological epigenome” associated with AD [116, 117]. Epigenetic mechanisms, such as modifications of DNA-structure or of associated histones, regulate gene transcription and may contribute substantially in the interplay of genetic and environmental factors in the determination of the human phenotype [118–120]. Alterations of the levels of 5-mC and 5-hmC and other epigenetic marks during the lifespan have been associated with the progression of AD. To date, several groups [121–123] have identified, by epigenome-wide analysis, several genes regulated by DNA methylation in human brain AD samples. These studies reflect that AD, as well as dementias in general, has specific epigenetic signatures [124]. In particular, several research teams have described age-dependent methylation changes of a number of AD-related genes. APP gene expression is partially regulated through methylation of the multiple CpG sites of its promoter, and hypomethylation events have been described in association with AD in patients over 70 years of age [125–127]. However, these data could not be confirmed by another study with bigger cohorts [128]. Other APP related genes, such as BACE1 (that codes for an enzyme responsible for the misprocessing of APP towards toxic Aβ generation), can be regulated via epigenetic mechanisms and hypomethylation states of that promoter have been associated with AD [129, 130]. Another enzyme associated with Aβ, PSEN1, has also been shown to have an aberrant methylation status in AD [129]. Importantly, Aβ itself has been described as an epigenetic modulator by inducing global DNA hypomethylation and specific hypermethylation of Neprilysin, an enzyme associated with its degradation [131], thus reducing its expression. Therefore, despite still not being well defined, it seems that the Aβ generation mechanisms are associated with DNA methylation patterns in a bidirectional manner. Tau protein, the other major pathomolecular change in AD, has also been shown to be regulated epigenetically. DUSP22 is a phosphatase with the ability to dephosphorylate abnormal tau and is downregulated in AD brain samples by hypermethylation of its promoter [117].

Besides the principal molecules associated with AD, several studies have indicated the importance of epigenetic processes in gene expression regulation that occur in AD. SORBS3, or Vinexin, encodes for a cell adhesion protein involved in synaptic function, and several groups have found a hypermethylation of its promoter [116, 132]. This process normally appears in an age-dependent fashion but is accelerated in AD. Other genes such as the gene NF-kB or some regions of the promoter of the gene Cyclooxygenase-2 (COX-2), both proinflammatory and associated with inflammatory events in AD, have been reported to be hypomethylated [133, 134]. Hypermethylation of the promoters of BDNF and cAMP response element-binding protein (CREB) were found in the frontal cortex of AD patients [133]. Both proteins are critical for neuronal survival [135, 136] and have been associated with AD [137]. The importance of the CREB signalling in AD is evident since other authors have also described alterations in genes related to this molecular pathway, such as hypermethylation of Thromboxane A2 Receptor (TBXA2R) [116], a G-protein receptor regulating CREB [138]. Recent epigenome-wide association studies (EWAS) identify another gene called Ankyrin1 (an adaptor protein; ANK1) in hypermethylated state in cortex samples of AD patients [121, 122]. Importantly, the epigenetic state of ANK1 showed a strong correlation both with early and late stages of the disease, suggesting its possible validity as a biomarker.

Besides DNA methylation, the role of histone modifications has been also dissected in AD [5]. However, few studies have focused on human brain samples. Of all the histone modifications described so far, lysine acetylation and lysine methylation are the most common [139]. For example, histone acetylation has been described to be reduced both in the human brain tissue and in AD mouse models [140, 141]. Importantly, the transcriptional activity of AD associated genes has been associated with specific histone marks, such as increased acetylation of H3 at the BACE1 promoter [130]. This histone mark activates gene transcription by relaxing the chromatin structure. One of the proteolytic fragments of APP is the APP C-terminal fragment (AICD). Several studies have demonstrated the ability of AICD to recruit, directly or indirectly, the histone acetyltransferase TIP60 [142, 143]. The consequences of this interaction are the repression of Neprilysin expression in NB7 cells by acetylation of lysines on histone H4 [144]. Also in human samples, other histone marks were found to be altered. For example, an increase of phosphorylation of histone H2AX, a histone variant, in the hippocampus was found in AD samples [145], as well as increased global H3 phosphorylation in the frontal cortex [133] and hippocampus [146].

However, most current knowledge on the involvement of the histone code derives from work done using transgenic mouse models. Decreased levels of H4 acetylation were found in APP/PS1 of the mouse model hippocampus after a learning task [147]. However, deeper analysis showed an increased H4 acetylation in the CA1 region of hippocampus together with increased H3 acetylation and phosphorylation in the prefrontal cortex of another APP model, the Tg2575 [148]. These data emphasize the necessity of restricting epigenetic analysis to small brain areas or even single-cell analysis to completely understand the role of the epigenetic processes in AD. H3 and H4 acetylation was increased in neuronal cell cultures from an AD mouse model expressing mutations in APP and in Tau (3xTg) [149]. Regarding other hallmarks of AD, hyperacetylation of H3 on the promoter of BACE1 in 3xTg brains [130] leading to increased transcriptional activity of the gene, as well as on the promoter of BACE1 and PS1 in N2a cells expressing a mutated form of APP, has been described [150]. Other modifications have not been sufficiently studied although experiments in animal models have explored histone methylation [151, 152] and ubiquitination [153] involvement in learning and memory processes, also indicating possible implications in cognitive impairments.

Although the AD “histone code” remains to be deciphered, it is evident that histone alterations play important roles both by altering histone marks and by changing the levels of histone modifying enzymes [141, 154] in dementias and are suitable targets for pharmacological approaches.

Non-coding RNAs [155] have also been associated with AD [156]. Several studies have analysed alterations of miRNA expression in several areas of post-mortem AD brains, showing a broad spectrum of changes in a multitude of miRNAs. Some of the most relevant changes occur in miRNAs targeting mechanisms implicated in APP and/or its misprocessing towards the amyloidogenic pathway. miR-106b [157] and -153 [158] are downregulated in AD (temporal cortex and frontal cortex, respectively), and one of its multiple targets is the mRNA of APP [159]. Other miRNAs with the ability to regulate APP are miR-101 [158] and miR-124 [160], and both are described as downregulated in AD brains. miR-137 and -181c are also downregulated in AD [161, 162], and their downregulation promotes APP processing into neurotoxic forms of Ab. Other key molecules of the amyloidogenic cascade like BACE1 are also targeted by several miRNAs. Numerous miRNAs with the capability of reducing BACE1 levels were found to be reduced in several areas of AD brains, for example the miR-29a/b-1 cluster, -29c, -107, -339-5p and -485-5-p [163–168]. Other ncRNAs also target BACE1, including the long ncRNA BACE1-AS, by regulating BACE1 RNA stability. BACE1-AS was described as being in an upregulated state in AD brains samples suggesting its role in incrementing BACE1 levels [169]. Tau is also regulated via miRNAs. miR-219 was found downregulated in the AD human frontal cortex [170] and is thought to regulate tau mRNA directly. Another enzyme involved in the aberrant phosphorylation of tau is Glycogen Synthase Kinase 3β (GSK3β) that is considered the major modulator of tau phosphorylation in the brain [132]. Additional studies have demonstrated that GSK3β is negatively regulated by miR-26a, a microRNA deregulated in AD [161, 171]. Several miRNAs impact on several hallmarks of AD simultaneously. miR-107 is another miRNA targeting BACE1 [166], but it is also capable of deregulating cdk5 [172], a kinase related to tau phosphorylation. In the case of miR-124 and -137, both target APP metabolism as well as the differential splicing of tau [161, 173]. miR-9 and miR-132 can also regulate tau splicing mechanisms [174–176]. Interestingly, both also have the ability to reduce SIRT1, a sirtuin that can deacetylate tau [154, 177]. Together with those, miR-34c and miR-181c can reduce the levels of SIRT1 and all of them are reduced in AD [162, 178, 179].

Another important alteration in AD is synaptic dismantling and alteration of synaptic transmission [180, 181]. While several miRNAs have been associated with those mechanisms [182], miR-132 and miR-125b in particular have been associated with AD. It has been described that miR-132 is reduced in the hippocampus, cerebellum and medial frontal gyrus, whereas miR-125 is increased in these areas [161, 174, 183]. Another ncRNA, the long non-coding RNA 17A, is elevated in AD brains and regulates GABA transmission [184]. Due to the role of microRNAs in synaptic plasticity [185] and increasing evidence that dysregulation of miRNAs biogenesis is implicated in AD, these epigenetic effectors seem critical not only in the normal gene expression pattern of neurons but also in the pathophysiology of AD. The complexity of ncRNA function and their intricate patterns of expression in nervous system demand further investigation, which may eventually lead to the discovery of new druggable targets to delay or prevent AD.

### AD’s pharmacoepigenomics

Overall, it is evident that epigenomic alterations in AD make suitable targets for therapeutic interventions (Table 2). However, so far, only histone alterations have been properly studied in pharmacoepigenomics.

The most common histone modifications (acetylation and methylation) are performed by the balanced activity of HATs and HDACs on the one hand and histone methyltransferases and demethylases (HDMTs) on the other hand [153, 186]. Due to the availability of drugs targeting those enzymes, most research performed to describe its role has been performed in mouse models of AD. The induction of histone acetylation through inhibition of HDACs has been proposed as a candidate approach to treat AD based on of several lines of evidence using such models [187]. One of the first demonstrations of the role of the potential use of HDACi to treat AD showed that administration of the unspecific HDACi sodium butyrate in an AD mice model (CK-p25 mice) was able to restore cognitive capabilities initially decreased in the transgenic mice [188]. An increased expression of HDAC2 in human AD samples (hippocampal area CA1 and entorhinal cortex) and also in the hippocampal area CA1 and in prefrontal cortex of the CK-p25 mice and the 5XFAD model has been described [141], reinforcing the idea of using HDACi to treat AD. Administration of another HDAC inhibitor TSA also improved memory formation in APP/PS1 mice by increasing H4 acetylation in brain [147]. VPA, another inhibitor of HDAC1, was useful in order to reduce Aβ levels and plaques in the hippocampus of an APP model (PDAPP) [189] and also was able to improve learning capabilities of an AD mice model. Similar data was obtained in a Tg2576 model treated with the HDAC inhibitor sodium phenylbutyrate, where chronic treatment reduced tau hyperphosphorylation but could not revert Aβ accumulation [190]. In subsequent studies, the same team treated younger animals with the same compound and found decreased Aβ accumulation and reduced immunoinflammatory events [190] indicating the importance of the disease stage chosen for treatment. Other drugs, such as SAHA, also improved cognitive capabilities of APP/PS1delta9 mice [191], and MS-275, a specific HDAC1 antagonist [192], showed the same improvement in cognition of APP/PS1 mice together with a reduction of amyloid plaques in the hippocampus of treated animals [193]. Although we still do not completely understand the role of HDAC inhibition in the brain [194], it seems evident that selective pharmacological inhibition of some of the multiple HDAC members is a promising area of research for treating early stages of AD.

## Conclusions

The involvement of epigenetic factors as key players in the ageing process in the brain and in age-related neurodegenerative and psychiatric disorders is widely accepted and provides important insights as to how they can potentially mediate interactions between genetic and environmental risk factors. In spite of epigenetic-based therapy emerging as an appealing alternative approach to the treatment of neuropsychiatric diseases with deregulation in multiple signalling pathways, many unresolved questions still hinder the progression of candidate therapies to clinical trials.

Future translational research approaches to the development of epigenetic therapeutics in neuropsychiatric diseases must overcome a number of limitations. One of the first bottlenecks is the heterogeneity in the design of studies that frequently use different experimental models, as well as in the particular regions of the brain analysed and in the variable sensitivity and resolution of the epigenetic methods employed. Most studies have been performed with small sample sizes and thus have low statistical power and have only addressed a few epigenetic marks in a few specific tissues. With this level of heterogeneity, it is very difficult to infer broad conclusions about the implications of epigenetics in neuronal development and its alterations in neuropsychiatric diseases. It is also important to note that studies in living humans, as opposed to those in in vitro and animal models, are very scarce. Other limitations regarding the design of the studies are the phenomenological and dichotomous definitions of the disorders, the multiple clinical manifestations, the inability to control lifestyle factors and the inability to distinguish chronological correlations between environmental exposure, epigenetic modifications and disease progression.

In order to address many of the questions mentioned above and increase the reproducibility of existing epigenetic findings, there are several challenges that must be confronted. Some important ones include the following: (1) to carry out larger, longitudinal, multicenter and prospective studies in order to investigate brain diseases and their interactions; (2) to consider tissue and cell-type specificity by using dissection of brain tissues; and (3) to include multiple epigenetic marks, genome-wide studies and integrate the results into specific chromatin contexts.

We must also consider the new challenges in epigenetic research. There is no doubt about the importance of non-coding RNAs in post-transcriptional regulation of gene expression in neural differentiation and their deregulation in several human disorders, including neuropsychiatric diseases [5, 195]. Furthermore, we must add new layers of complexity such as the epigenetic regulation of RNA (i.e. RNA methylation) [196], three-dimensional chromatin structure as a key regulator of transcription [197] and the epigenetic control of the mitochondrial genome that can explain the mitochondrial dysfunction observed in neuropsychiatry diseases [198].

Emerging technologies for epigenetic research can also improve our knowledge. As one prominent example, the use of CRIPSR/Cas9 technology and its adaptations to different models (for example, epigenetic editing) can demonstrate the causal role of epigenetics in instructing gene expression [16]. In addition, computational modelling can accelerate the search for new epigenetic therapeutic approaches to treat neurological disorders, map them to clinical predictions and further our understanding of complex brain diseases at the individual and population levels [199]. In light of the latest advances in induced pluripotent stem cell (iPSC) technology, future epigenomic brain approaches will involve the study of specific neuronal populations derived from patient-cells, allowing a better understanding of the disorder by disease modelling and a faster drug screening/repurposing in a personalized manner [200].

It is clear that current knowledge of the epigenetic changes that occur during healthy ageing and pathological conditions in the brain is increasing, but much research is still required before translating the findings to clinical practice. This is of particular relevance due to the numbers of elderly people in third world societies and the social effects of cognitive impairment. In summary, we need to overcome important challenges to identify new epigenetic therapeutic targets and to develop appropriate, randomised and controlled trials with human subjects.

## Abbreviations

5-hmC: 5-Hydroxymethylcytosine
5-mC: 5-Methylcytosine
AD: Alzheimer’s disease
APP: Amyloid precursor protein
DG: Dentate gyrus
DNMT: DNA methyltransferase
EOAD: Early-onset Alzheimer’s disease
FAD: Familiar Alzheimer’s disease
HAT: Histone acetyltransferase
HDAC: Histone deacetylase
HDACi: Histone deacetylase inhibitor
HDMT: Histone demethylase
HMT: Histone methyltransferase
LOAD: Late-onset Alzheimer’s disease
ncRNAs: Non-coding RNA
NSPCs: Neural stem progenitor cells
PTSD: Posttraumatic stress disorder
SAHA: Suberoylanilide hydroxamic acid
SGZ: Subgranular zone
SVZ: Subventricular zone
SZ: Schizophrenia
TET: Ten-eleven translocation
TSA: Trichostatin A
VPA: Valproic acid
